# Gravity and scaling laws of city to city migration

**DOI:** 10.1371/journal.pone.0199892

**Published:** 2018-07-06

**Authors:** Rafael Prieto Curiel, Luca Pappalardo, Lorenzo Gabrielli, Steven Richard Bishop

**Affiliations:** 1 Department of Mathematics, University College London, Gower Street, WC1E 6BT, London, United Kingdom; 2 Istituto di Scienza e Tecnologie dell’Informazione (ISTI), Consiglio Nazionale Delle Ricerche (CNR) - Area della Ricerca di Pisa, Via G. Moruzzi, 1, 56127, Pisa, Italy; 3 Department of Computer Science, University of Pisa, Largo Bruno Pontecorvo 3, 56127, Pisa, Italy; Purdue University, UNITED STATES

## Abstract

Models of human migration provide powerful tools to forecast the flow of migrants, measure the impact of a policy, determine the cost of physical and political frictions and more. Here, we analyse the migration of individuals from and to cities in the US, finding that city to city migration follows scaling laws, so that the city size is a significant factor in determining whether, or not, an individual decides to migrate and the city size of both the origin and destination play key roles in the selection of the destination. We observe that individuals from small cities tend to migrate more frequently, tending to move to similar-sized cities, whereas individuals from large cities do not migrate so often, but when they do, they tend to move to other large cities. Building upon these findings we develop a scaling model which describes internal migration as a two-step decision process, demonstrating that it can partially explain migration fluxes based solely on city size. We then consider the impact of distance and construct a gravity-scaling model by combining the observed scaling patterns with the gravity law of migration. Results show that the scaling laws are a significant feature of human migration and that the inclusion of scaling can overcome the limits of the gravity and the radiation models of human migration.

## Introduction and background

Today, human migration is one of the most debated concerns to the general public, governments and international agencies, due to the importance of integration policies, socioeconomic development and well-being. On the one hand, migrants contribute to the prosperity of their destination, to which they provide new skills, norms and community activities, as well as easing the pressures of an ageing population [1] and can enhance conditions in their place of origin by either reducing unemployment, improving conditions by sending remittances which reduces poverty and increases the resilience in the case of disasters [2, 3]. On the other hand, human migration creates the political challenge of designing integration policies to allow newcomers to settle in unfamiliar environments, as well as prompting the need for improvement of social support systems [4, 5]. Understanding, modelling and predicting human migration is thus of fundamental importance for the formulation, planning and implementation of balanced policy programmes.

It is therefore not surprising that the study of human migration has attracted the interest of scientists from many disciplines. Some studies investigate the dynamics of specific types of migration, such as international migration [6, 7], migration from rural to urban areas [8, 9], mobility in urban areas [10–12], or disaster-, climate change- and conflict-induced migration [13–18]. Other studies focus on different models of migration, such as models based on a Markov process [19–21] or the cumulative inertia model, according to which an individual is less likely to migrate if they spend more time in the same place [22]. Two prominent migration models are the gravity model, which considers both the population size of the places of origin and destination and the distance between them [6, 23], and the radiation model, which additionally takes into account job opportunities in the vicinity of the place of origin [24].

Migration between cities, as well as from the countryside to the cities, has attracted particular interest in recent years. Internal migration is indeed the main reason for urban growth [1] and the reason why most of the world’s population now live in urban areas. A city’s population size strongly affects the well-being of its inhabitants [12, 25, 26], as large cities provide more efficient resources for their inhabitants [27], who tend to develop more social contacts [28], move in a more diversified way [12, 26] and create more patents and bank deposits [27]. On the negative side however, large cities suffer more infectious diseases [27] as well as more crime [29, 30]. Migration is the main driver of city changes [31] and the reason why some cities grow faster than others, providing a positive feedback leading to even more changes and further population growth [32]. An important challenge is to understand, quantify and predict the impact of city size on human migration: are individuals in large cities more likely to migrate than individuals who live in small towns? Are individuals more likely to migrate to a city larger than their current one, or does population size not matter? Can we quantify the attractiveness of a city for internal and international migrants based on its population size?

Here, we study human migration in the context of cities and its population [33]. We analyse migration dynamics considering individuals as the inhabitants of a city (or potential movers to them) [34] but ignoring other individual aspects, such as age, income, education or gender. We consider a *migrant* as an individual who permanently moves from one location to another, so not considering movements within the same urban area, regardless of their legal status and without positive or negative connotation. Starting from official census data, we analyse the migration fluxes from and to US cities and investigate how these fluxes are influenced by the cities’ population size. Our main finding is that migrants preserve city size, i.e., they prefer to migrate to cities with a similar size to the city of their origin. Moreover, we observe a phase transition where the exponent in our model changes from sublinear to superlinear at a specific population size. Building upon these findings we develop a data-driven scaling model which describes human migration as a two-step decision process, demonstrating that it can partially explain migration fluxes only on the basis of city size. We then consider the impact of distance on a gravity-scaling model of human migration, showing that it performs better than the scaling, gravity and radiation model of human migration. The code of the models is available online: https://github.com/rafaelprietocuriel/Migration.

### Migration data

Our data source is a census which stores the number of migrants from one metropolitan area to another in the US [35], where a metropolitan area or *city* is considered here as a high population density area with strong economic ties and with a population larger than 50,000 inhabitants. It is based on the metropolitan statistical areas (MSAs) defined by the U.S. Department of Commerce. This gives 385 cities for which the internal migration process is quantifiable (the area of Los Angeles was merged from the original data source with other three metropolitan areas: Riverside-San Bernardino, Oxnard-Thousand Oaks-Ventura and Bakersfield). These cities are collectively formed by approximately 268 million inhabitants, so more than 80% of US population. The population size of individual US cities varies broadly from just above 50,000 inhabitants (e.g., Carson City) to nearly 20 million inhabitants (New York City and Los Angeles) while individuals living in towns or rural areas with less than 50,000 inhabitants are considered to be part of the *countryside*. Using the available data, we analyse the following aspects of city to city migration:

1. the probability that an individual chooses to migrate (Section *To migrate or not*);
2. the destination picked by migrants according to the size of their city of origin (Section *Migration to and from other cities*);
3. the probability that an individual moves to the countryside (Section *Migration to and from the countryside*);
4. the destination picked by international migrants (Section *Migration to cities from another country*).

## Scaling of migration

We assume that there are *n* cities and in the following we define *X*_*ij*_ as the number of individuals migrating from city *i* to city *j*. We define *X*_i*_ as the (total) outflow migration from *i* and X_*j_ as the (total) inflow migration to *j*, such that ∑_*j*_ *X*_*ij*_ = *X*_i*_; and ∑_*i*_ *X*_*ij*_ = X_*j_, and *P*_*i*_ denotes the size of the population living in city *i*, with *i* and *j* = 1, 2,…,*n*.

### To migrate or not

We estimate that the probability of an individual deciding to migrate from city *i* as *X*_i*_/*P*_*i*_, which is the frequency of a resident leaving city *i*. We investigate how this probability depends on city size by fitting a power law equation:

$$X_{i*}=\alpha P_{i}^{\beta },$$ (1)

where *α* and *β* are parameters to be determined from the data (and then expressed as $\hat{\alpha }$ and $\hat{\beta }$, respectively). Eq 1 is a functional form that does not assume that the probability of migrating either increases or decreases with city size. Rather, this is a data-driven model so that the data provides evidence supporting whether the probability of migrating increases with city size, if $\hat{\beta }>1$, referred to as superlinear [25], decreases, if $\hat{\beta }<1$, referred to as sublinear, or if it is independent, if $\hat{\beta }$ is close to one.

We fit the exponent $\hat{\beta }$ from the dataset and find a sublinear behaviour of the probability of migrating, with $\hat{\beta }=0.8829\pm 0.0147$, i.e., the probability that an individual moves away from their city decreases as the size of the city increases. Moreover, we observe a coefficient of $\hat{\alpha }=0.1676$, indicating that the probability of migrating from a city ranges between 0.023 (as for New York City or Los Angeles) and 0.047 (for instance, in Carson City in Nevada or Victoria in Texas). Our results indicate that individuals from the smallest cities (say, with less than 100,000 inhabitants) are twice as likely to migrate than individuals from cities with more than 10 million inhabitants.

Patterns of human migration are quite variable among cities of different sizes and so noise is a relevant issue. One of the key aspects to consider is that 73% of the cities in the US have a population smaller than 500,000 inhabitants, and 50% of the cities have a population smaller than 250,000 inhabitants. Therefore, most of the observations are actually small cities, for which a large variation is expected, as we are comparing, for instance, the number of individuals who move into Yuma, Arizona, a city located in the border between the US and Mexico with the number of individuals who move into Rochester, Minnesota, a city in which the Mayo Clinic forms its core economy. Both cities have approximately the same population but their migration patterns might have different push and pull factors. However, by observing the average small city and comparing its migration patterns to medium-sized or large-sized cities, a different average pattern is observed, and these differences are, in most cases, statistically significant. The scaling equation detects a generalised pattern but it does not mean that all individuals from smaller cities have a higher probability of migrating than individuals from large cities. When Eq 1 is fitted, the adjusted *R*^2^ is 0.9033, meaning that there are other aspects which determine the individual probability of migrating (for instance, age) which in turn determine the collective frequency of migrating from each city. However, a general pattern in which individuals from smaller cities are more likely to migrate is, nonetheless, detected.

### Migration to and from other cities

Having decided whether or not to migrate, the decision to migrate to a particular city of a given size is also affected by the population size of the origin city. For instance, if we select only individuals who used to live in a small city (say with 50, 000 ≤ *P*_*i*_ ≤ 200, 000) and fit Eq 1 looking at the size of the cities to which they migrated, we find a sublinear behaviour with $\hat{\beta }=0.8060\pm 0.0263$ and adjusted *R*^2^ = 0.7101. We find a similar sublinear behaviour when using a different “small city” threshold, for instance, for individuals who used to live in a city with less than 500,000 inhabitants ($\hat{\beta }=0.8224\pm 0.0206$ with adjusted *R*^2^ = 0.8061), less than one million inhabitants ($\hat{\beta }=0.8363\pm 0.0175$ with adjusted *R*^2^ = 0.8554) or other thresholds within that range (see the section Tables of results for the table of coefficients).

In contrast, we find a superlinear behaviour (*β* > 1) when fitting Eq 1 but considering only the destination of individuals who used to live in “large cities” and decided to move, that is, if we select only individuals who used to live in cities with a population larger than a certain threshold. For instance, for *P*_*i*_ ≥ 5 million, we find a slight superlinear behaviour, as $\hat{\beta }=1.0499\pm 0.0337$, with adjusted *R*^2^ = 0.7163. This behaviour gets more pronounced with a larger threshold, so we find that $\hat{\beta }=1.1688\pm 0.0506$ (with adjusted *R*^2^ = 0.5814) with *P*_*i*_ ≥ 8 million and that $\hat{\beta }=1.2984\pm 0.0619$ (with adjusted *R*^2^ = 0.5327) with *P*_*i*_ ≥ 10 million (see the section Tables of results). This means that an individual who used to live in a large city (i.e., *P*_*i*_ > 5 million) is more likely to move to an equally large city than to move to a small city. Thus, individuals tend to preserve city size when deciding to migrate: an individual from a city with several million individuals is almost twice more likely to move to a city with several million individuals as compared to an individual from a small city and similarly, individuals from the smaller cities are more likely to move to equally small cities.

Migration patterns can also be analysed in terms of the influx of population into a city, interpreted as the arrival of people per 1,000 inhabitants. Although we have found that individuals who live in large cities are more likely to move to a large city the next year, that does not necessarily mean that the influx of people who arrive into a large city come from equally large cities since it depends on the distribution of city size. Thus, by fitting the power law equation

$$X_{*j}=\alpha P_{j}^{\beta }$$ (2)

we now consider the inflow of people who move to city *j*.

Again, if we consider only the influx of individuals who move to a city with population in the range 50, 000 ≤ *P*_*i*_ ≤ 200, 000 (i.e., a “small city”), we find a sublinear behaviour with $\hat{\beta }=0.7997\pm 0.0299$ (with adjusted *R*^2^ = 0.6492) and find a similar sublinear behaviour when using a different population range, for instance, for the influx of individuals who move to a city of less than 500,000 inhabitants ($\hat{\beta }=0.8159\pm 0.0245$ with adjusted *R*^2^ = 0.7429). In general, the impact of the sublinear behaviour gets more pronounced (that is, $\hat{\beta }$ gets much smaller than one) for intervals with smaller cities (see the section Tables of results for the table of coefficients).

In contrast, we find a superlinear behaviour if we look at the influx of individuals who move to a “large city”. For instance, if we analyse the influx of individuals who moved to a city with more than 8 million people, we find a superlinear behaviour with $\hat{\beta }=1.1180\pm 0.0460$ (with adjusted *R*^2^ = 0.6053) and similarly if we look at the influx of individuals who moved to a city with more than 10 million inhabitants with $\hat{\beta }=1.2539\pm 0.0574$ (with adjusted *R*^2^ = 0.5538). Roughly speaking, 1.7 individuals in every 1, 000 inhabitants of a city with millions of individuals (such as Los Angeles) will have lived in a small city during the previous year, but nearly 20 individuals in every 1,000 in a small city will have lived in a different small city the previous year.

### Migration to and from the countryside

An individual who lives in a city might decide to migrate to the countryside and this decision is affected by the size of the origin city. By fitting a power law equation (Eq 1) we find that an individual who currently lives in a city might decide to move to the countryside and, according to the data, the probability of moving has a sublinear behaviour, with ($\hat{\beta }=0.6846\pm 0.0273$, $\hat{\alpha }=0.7214\pm 0.3464$ and with adjusted *R*^2^ = 0.6199). Thus, results show that an individual who lives in a city with less than 200,000 people is four times more likely to move to the countryside than an individual who lives in a city with 20 million inhabitants, such as Los Angeles or New York City.

Also, an individual who currently lives in the countryside might decide to move to a city and a scaling pattern for the size of their destination follows a sublinear behaviour ($\hat{\beta }=0.5971\pm 0.0342$, $\hat{\alpha }=0.4299\pm 0.4331$ and with adjusted *R*^2^ = 0.4421). Thus, an individual who currently lives in the countryside is 6 times more likely to move to a city with 200,000 inhabitants or less than to a city with 20 million individuals.

### Migration to cities from another country

We find that the destination of international migrants is also affected by the city size of the destination. An individual who arrives in the US from another country is more likely to move to a large city, that is, international migration also exhibits a superlinear behaviour. Larger cities in the US increase their population diversity, measured simply as the proportion of individuals who previously lived outside the US [36], three times faster than smaller cities, ($\hat{\beta }=1.1884\pm 0.0339$, with adjusted *R*^2^ = 0.7610) with an even more pronounced pattern for individuals from Africa ($\hat{\beta }=1.5794\pm 0.0728$, with adjusted *R*^2^ = 0.5500) and Americas outside the US ($\hat{\beta }=1.2808\pm 0.0424$, with adjusted *R*^2^ = 0.7036).

The inflow of international migrants for every 1,000 inhabitants varies according to the size of their destination. Thus, comparing the whole range of city size, we observe that large cities with millions of people are increasing their percentage of the population from Africa and from Americas outside the US, 32 times and 5 times faster respectively than the smallest cities (see the section Tables of results for the full table of coefficients).

### Migration patterns

Fitting a scaling equation and considering the destination of individuals who lived in small cities results in a sublinear scaling pattern in terms of their probability of moving and their destination, whether we consider small to be cities with less than 200,000 inhabitants or even less than 1 million. Similarly, considering only individuals from the “large cities”, where the term “large” can be cities with more than 6 million people or more, gives a superlinear result in terms of the destination picked by its migrants. Thus, there is a phase transition between a sublinear behaviour for small cities to a superlinear behaviour in the case of large cities and, in general, this pattern tends to get more pronounced at the extreme values of city size, that is, $\hat{\beta }$ gets smaller for the smallest cities and larger for the largest cities. Additionally, by considering the inflow of migrants, we observe a sublinear pattern for small cities and a superlinear pattern for the large cities. Thus, there is also a transition for the influx of migrants into a city.

**Fig 1:**
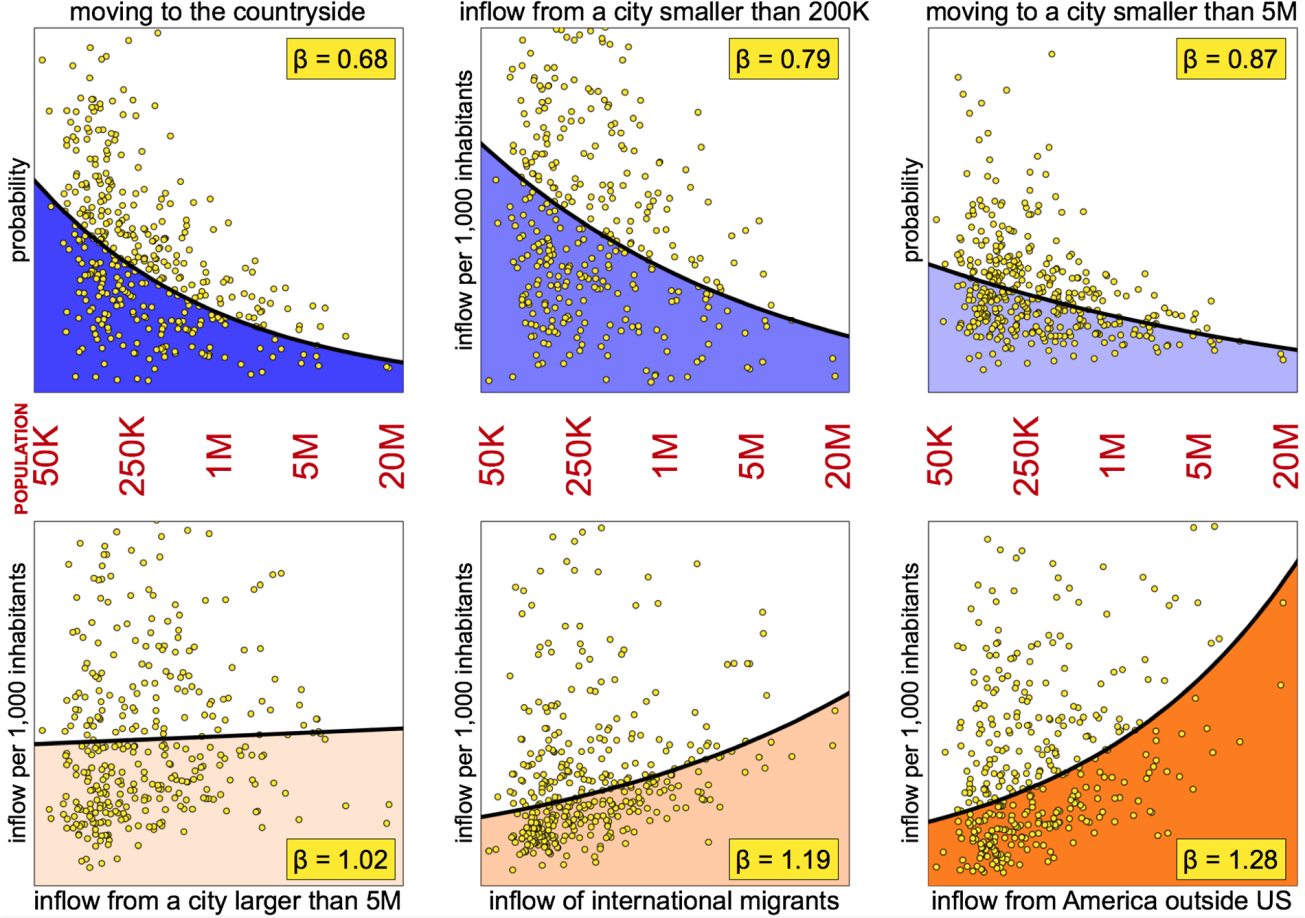
Selected scaling relationships fitted to the data. The dots represent data on each of the 385 cities in the US with its size on the horizontal axis and its corresponding figures for migration given on the vertical axis given in different units (as a probability or as the inflow of migrants per 1,000 inhabitants). Also plotted on the same diagrams are the results of the scaling relationship fitted to the data with the coefficient $\hat{\beta }$ given in each case. Top panel: three sublinear relationships. Bottom Panel: three superlinear relationships. A coefficient $\hat{\beta }\approx 1$, as establish in the diagram on the left in the bottom panel (for the inflow from a city larger than 5 million), means that city size has negligible impact on that flux. This establishes the approximate city size where a phase transition occurs.

Migration patterns, either the decision to move to another city, move to a small city given that the individual lives in an equally small city, the inflow of individuals who move from another city or from another country are all influenced by city size, either the size of the origin or the destination city and some of the patterns presented here are sublinear and some superlinear (Fig 1). The observed phase transition occurs roughly for cities between 1 and 5 million inhabitants. Below that, cities follow a sublinear pattern and above that, cities follow a superlinear pattern in terms of the destination picked by migrants. Detecting a sublinear pattern in the destination picked by migrants required grouping cities with a population smaller than a certain threshold and to analyse the observed pattern from the whole group. Thus, it is by grouping cities with a similar population size that we are able to detect an emergent pattern.

**Fig 2:**
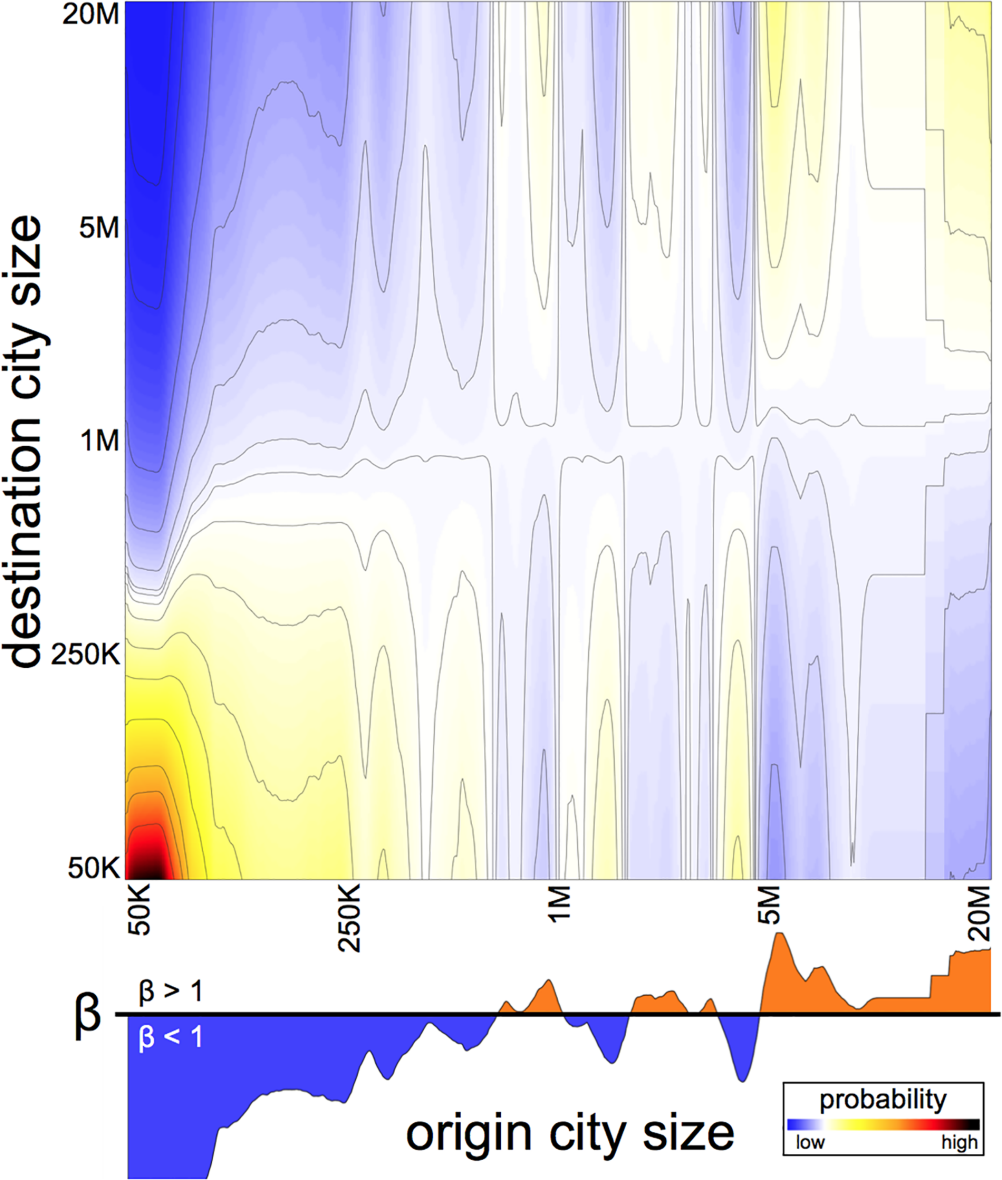
Probability of migration conditional on city size. Probability of migrating from a city of a given size (horizontal axis) to a city of a given size (vertical axis). The fitted values of $\hat{\beta }$ according to the city size (plotted in the lower panel) indicates if the probability of migrating to a destination with a given size follows a sublinear ($\hat{\beta }<1$, in blue) or superlinear ($\hat{\beta }>1$, in orange) behaviour. For example, we observe that if the city of origin is larger than 4 million inhabitants, then the probability of migration follows a superlinear behaviour. In contrast, a strong sublinear behaviour is observed for small cities, particularly if the city has less than 100,000 inhabitants.

To analyse the migration data, not just for “small” or “large” cities, we designed an algorithm which takes a ranked list of the cities according to their size, using a logarithmic scale, and creates non-overlapping partitions using a moving window of various ranges and with a random starting point. This allows us to group similar cities in terms of their population size but varying what we mean by similar. The cities were then partitioned 1,000 times, each time considering a partition with a different starting point and a different width, such that on each partition, cities are grouped based on slightly different criteria. For instance, one partition might consider an interval *I*_1_ = 270, 000 ≤ *P*_*i*_ ≤ 355, 000 while another time cities might be partitioned in such a way as to create an interval *I*_2_ = 290, 000 ≤ *P*_*i*_ ≤ 390, 000. Thus, on each run of the partitioning process, particular cities might be grouped in different ways. Then, taking into account the destination picked by migrants from the different cities within each interval, we obtain the scaling coefficient $\hat{\beta }$ by fitting Eq 1 for each interval of cities. Intervals with no cities inside are ignored. The result after grouping 1,000 times the cities was roughly 33,000 intervals and so the scaling equation was fitted this number of times and then, for each point in the population range, its corresponding values of $\hat{\beta }$ were averaged. Finally, for each point in the whole population range, we obtained an estimated value of the $\hat{\beta }$ which smooths out any possible decision of considering different ranges of city size. The results of the $\hat{\beta }$ for each population range gives us a stable and consistent way of estimating the scaling pattern observed for the cities in the US (Fig 2).

The size of the destination, picked by an individual who chooses to migrate, depends on the size of the origin city. For example, the probability that an individual from a city with less than 100,000 inhabitants moves to a city with less than 100,000 inhabitants is 44 times larger than the probability that they will migrate to a city with 10 million inhabitants or more. The resulting relationship is thus given by a $\hat{\beta }$ coefficient which captures the probability of moving to a city with any size according to the size of the origin (Fig 2).

## Models for the dynamics of city to city migration

### Scaling model

City size plays a strong role in determining the patterns of city to city migration: from the decision of whether to migrate or not (sublinear), whether to migrate to the countryside (sublinear), move to a small city (sublinear), move to a large city (superlinear) and in the destination for international migration (superlinear). Eq 1 determines the estimated probability that an individual living in a city with population *P*_*i*_ migrates from one year to the next (given by $\alpha P_{i}^{\beta -1}$). Fig 2 shows the relationship between the city size and the frequency of migration by considering the probability of each destination, given that an individual actually migrates. These two relations fully determine the dynamics of migration between different cities.

To take individuals from the countryside (51 million people) into account within the model, we consider that with a probability $\hat{p}=0.0322$ an individual will migrate from the countryside to a city from one year to the next and their destination city follows a sublinear behaviour ($\hat{\beta }=0.5971\pm 0.0342$). Also, an individual who currently lives in a city might decide to move to the countryside with a sublinear probability ($\hat{\beta }=0.6846\pm 0.0273$). This also fully determines the dynamics of migration between the countryside and the different cities.

**Fig 3:**
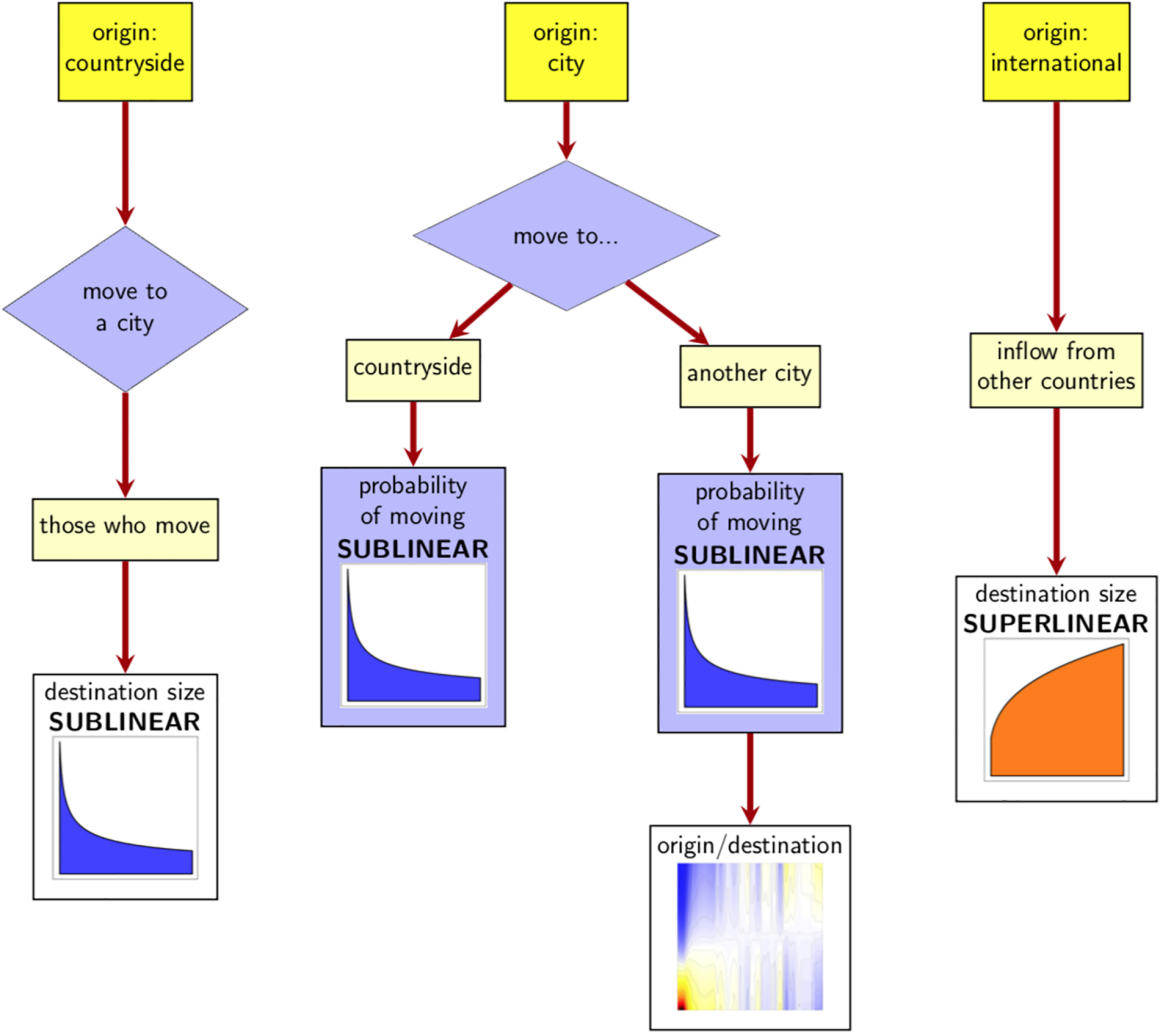
Schematic model of migration dynamics considering the location of an individual between two consecutive years. An individual from the countryside decides to migrate to a city from one year to the next one (with probability $\hat{p}=0.0322$) and the destination is chosen following a sublinear pattern. An individual from a city might migrate to the countryside (with a probability that decreases sublinearly with city size) or might decide to move to another city (with a probability that also decreases sublinearly with city size) although in this case, the destination is selected according to the city size of the origin and destination (Fig 2). Finally, an individual who arrives from another country picks their destination following a superlinear pattern according to city size.

We consider a two-step simulation for the dynamics of internal migration observed in the US with individuals moving between different cities or between the countryside and the various cities. We also assume the Markov property so that an individual’s choice to migrate, as well as their destination, are based only on the current location (that is, the size of their city for individuals who live in a city or the fact that they live in the countryside). In the first step, we simulate for each individual, whether they migrate or not, while in the second step we determine the destination of the ones who have chosen to move. Since both steps are affected by the observed scaling laws, we model migration as a decision problem [37]. Assuming no deaths or births and ignoring international migration (both arriving and leaving the US) the population dynamics is fully determined. The impact of city size in the migration pattern is summarised in Fig 3.

The observed scaling laws of city to city migration allow us to model the migration process by considering the distribution of US population living in different cities (83% of the US population) and the population living in the countryside (17% of the US population) and to consider the corresponding urban dynamics [33].

### Impact of distance and the gravity-scaling model

Undoubtedly, physical distance has an impact on human migration [37] which is not considered in the scaling model, so far. Thus, using only city size as a variable to determine the probability of migrating and the destination picked by those who actually move, we expect, for instance, roughly the same number of individuals moving to Los Angeles from Stockton-Lodi (a city in California with 684,000 inhabitants, located 500 kilometres away from Los Angeles) as those from Charleston (a city in South Carolina with 680,000 inhabitants, located 3,500 kilometres away from Los Angeles) simply because both, Stockton-Lodi and Charleston have (almost) the same population. However, data shows that there were 7.5 more people moving from Stockton-Lodi to Los Angeles than from Charleston. Physical distance is indeed relevant.

The *law of migration*, first published by Ernst Georg Ravenstein in 1885, developed looking at migration at county level from and to the UK, Ireland and Scotland, states, among many key issues [38], that the majority of migrants move a short distance. For more than a century there has been quantitative evidence that distance is one of the key aspects of migration and, in general, migration is inversely proportional to the distance between two locations.

As a consequence, one of the most commonly used models of human migration is called the *gravity model* (due to the similarity with the concept of physical gravity, in which objects are attracted to each other with a force directly proportional to their mass and inversely proportional to the distance between them) [6, 23]. The gravity model predicts the flux of migrants *F*_*ij*_ between locations *i* and *j* as:

$$F_{ij}=\frac{aP_{i}P_{j}}{d_{ij}^{b}},$$

where *a* is a constant which needs to be estimated from the data, *b* is a constant which takes into account the impact of distance and *d*_*ij*_ is the geographic distance between the two locations which in our case are cities, although the gravity model has been used to estimate the flux of migrants between countries, as for instance [7, 39]. Although the gravity model provides a valuable starting point for the analysis of migration, it has several drawbacks, for instance, it predicts the same flux from *i* to *j* as it predicts from *j* to *i*; it assumes a linear impact of the population of each location on the flux; in some cases it predicts more individuals leaving a location than the amount of individuals in the location and other issues (see [24]). There are some modified versions of the gravity model which remove the linearity or the symmetry of the flux [40] but one of the main issues to consider when using the gravity model is that it ignores any scaling factor and so all modified versions of the gravity model assume that individuals from small and large cities behave the same and have the same probability of migrating, as opposed to the results demonstrated earlier based on data.

To rectify this, a modified scaling model, a *gravity-scaling model for human migration* is constructed by modifying the destination picked by migrants. It takes into account the impact of distance and it also considers the scaling factor observed in the probability of migrating and the preferential destination picked by those who actually move. Consider an individual from city *i*, with population *P*_*i*_ who has decided to migrate. According to the scaling model (Fig 3) the probability that the individual moves to city *j*, say *π*_*ij*_, follows a scaling pattern with some *β* (which could either be greater than one, if *i* is a large city, smaller than one if *i* is a small city or close to one if *i* is near the phase transition, according to Fig 2). We consider the modified probability of moving from city *i* to city *j*, $\pi_{ij}^{\prime }$ as

$$\pi_{ij}^{\prime }=C\frac{\pi_{ij}}{d_{ij}},$$

where *d*_*ij*_ is the geographic distance between cities *i* and *j*, and *C* > 0 is a number which makes the set of probabilities $\pi_{ij}^{\prime }$ sum to one. Although other expressions of the gravity model of migration consider the impact of the distance squared, or other functions of the distance, not necessarily linear (for instance [24, 39, 40]), here we simply assume that the probability that the individual will migrate between two cities decreases as the distance between them increases. Note that the fact that it is a set of probabilities (i.e., they have to sum to one) means that the distance causes a non-linear impact.

The gravity-scaling model takes into account the observed scaling probability that an individual will migrate as well as the preferential migration observed between individuals from small o large cities and the impact that the physical distance has on the migration patterns. The gravity-scaling model gives, in our case, the same results as the scaling model for the migration to and from the countryside and for the inflow of international migrants as the distance between a specific city and the countryside or a continent is not well defined.

## Results

The fit of the power law equation (Eq 1) is, in most cases, good (see the section Tables of results for the full table of coefficients), as expressed by the high adjusted *R*^2^ obtained from the data. To determine the validity of the results of the scaling model and the gravity-scaling model (as measured by how well they fit the observed data), we compare against the commonly used gravity model and the radiation model of human migration. The two parameters of the gravity model ($\hat{a}=2.59\times 10^{-6}$ and $\hat{b}=0.753$) were estimated by minimising the mean square error of the model and the estimated flow between every pair of cities given by the radiation model was computed using the population density of the US. The results of the scaling model and of the gravity-scaling model are obtained by simulating the model dynamics 100 times, considering 53.2 million people at each time (20% of the total urban population) who first decide whether or not to migrate and then choose the destination, both according to their city size. The median of the 100 simulations is reported.

Under the scaling model dynamics, 3.1% of the metropolitan population of the US migrates each year. Also, a random individual from the cities in the US lives in a city with 4.92 million people, but after migration, it is expected that they will live in a city with 4.81 million people. Ignoring births and deaths and international migration, 80.5% of the movers went to a city with less population than their origin.

We compare the observed migration between every pair of cities and the predicted migration by the gravity, radiation, scaling and gravity-scaling models and report the mean square error and the maximum error in absolute value in Table 1. Ignoring the impact of distance (as in the scaling model) shows large departures from the observed migration flows, but also, ignoring the scaling factor on migration (as in the gravity model and the radiation model) yields on large errors. The gravity-scaling model has the best results in terms of the fit of the migration flux (see Table 1).

**Table 1:** Results of the scaling, gravity, radiation and gravity-scaling models. Mean square error and maximum error comparing the migration flow considering all pairs of cities as origin and destination. The smallest mean square error and the smallest maximum square error (in absolute value) are provided by the gravity-scaling model.

model	mean square error	max error
scaling	102,112.4	15,547
gravity	82,278.8	25,929
radiation	71,463.5	21,758
**gravity-scaling**	**58,288.8**	**9,592**

**Fig 4:**
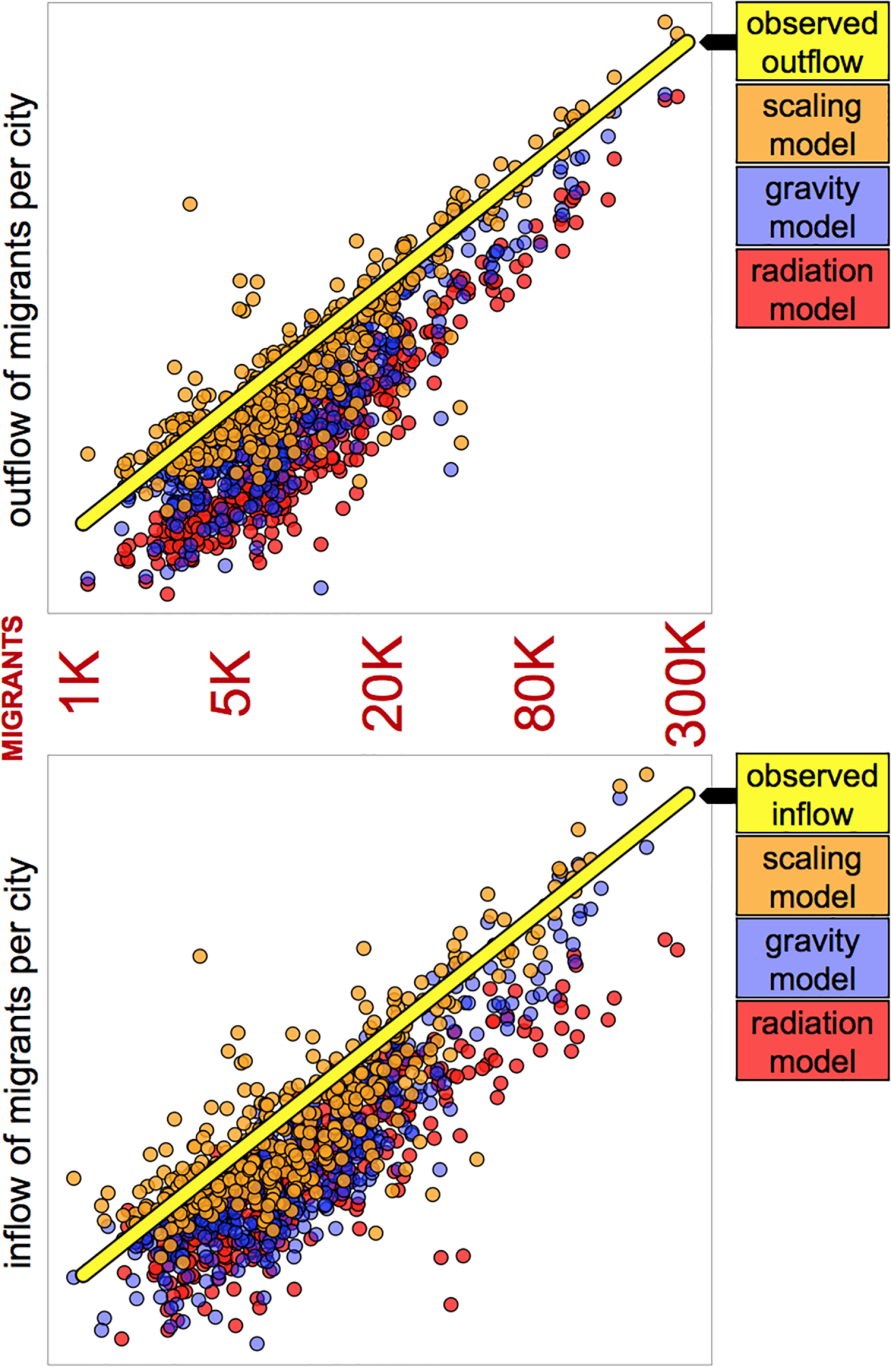
Observed outflow and inflow of migrants from each city against the predicted values of the scaling and the gravity model. The horizontal axis is the observed outflow or inflow of migrants from each city and the vertical axis is the results of the scaling and gravity models. The yellow line represents the identity (where the predicted value of the outflow or inflow of migrants from each city match the observed values, so there is a perfect match), so that observations closer to that line have a better fit. The gravity model (with blue colour) shows a systematic bias on the smaller cities.

Also, we compare the outflow and inflow of migrants from each city provided by the four migration models. Results show (see Fig 4) that the gravity model and the radiation model, as opposed to the scaling model, have a systematic bias and underestimate the outflow of migrants for the smaller cities (those with the smallest outflow of migrants), as it ignores the fact that individuals from small cities are more likely to migrate, as described by Eq 1. Also, both the gravity model and the radiation model underestimate the inflow of migrants to small cities and this is mainly because they also underestimate the outflow of individuals from small cities which have preferential migration to equally small cities. In general, the gravity model and the radiation model both have a systemic issue related to small cities, which is corrected by the scaling model. The gravity-scaling model, similar to the scaling model, takes into account the fact that individuals from small cities are more likely to migrate and so it does not present any systematic bias as the gravity model does. The comparison of the four models reveal that ignoring the distance between cities (as it is done by the scaling model) does not provide a better fit in terms of the mean square error. However, the scaling model does take into account that individuals from small and large cities behave differently and therefore does not have a bias, as opposed to the gravity model and the radiation model.

When determining the validity of the scaling and the gravity-scaling model, note that internal migration from and to the countryside and international migration should be also taken into account. The scaling model predicts that roughly 1.51 million individuals will move from the cities to the countryside and they will more frequently be from the smaller cities, whilst 1.64 million individuals will move from the countryside to a city and they are more likely to move to smaller cities. The destination picked by individuals who move from the countryside to a city has a sublinear behaviour: the scaling model predicts that less than 109,000 individuals from the countryside moving to the four largest cities of the US, simply because individuals from the countryside are more likely to move to small cities than large cities. Similarly, if we consider individuals who used to live in the countryside who then moved to the smallest 100 cities of the US, the gravity model predicts less than 67,000 movers, when in fact there were nearly 190,000 individuals moving. In this case, the scaling model predicts 156,000 movers, which is a much better fit.

International migration is also affected by city size. Although it is not possible to determine the impact of the size of the origin city and it is not possible either to compare against the gravity or the radiation model, the data does allow us to measure the scaling of international migration based on the destination. According to the scaling model, nearly 1% of the population of the largest cities lived in a different country the previous year, thus, increasing the diversity and multiculturalism of cities like New York, Los Angeles or Chicago. Large cities are increasing their diversity three times faster than small cities.

The scaling model is based on a set of observations in which noise is a relevant issue, so that we are detecting a generalised pattern, for instance, individuals from small cities have a higher probability of migrating, but it does not mean that all individuals from all small cities have a higher probability of migrating. The scaling model and the gravity-scaling model do not provide deterministic results. By simulating several times (100 in our case) under the same dynamics, both models provide natural departures which could be observed under the same dynamics. For instance, between Houston and Dallas, there were 14,666 migrants and results from the 100 simulations of the gravity-scaling model show that a migration flux between 14,485 and 15,745 is expected under the same dynamics.

## Conclusions

Power laws correctly describe many aspects of human activity, from the frequency of family names, the wealth of the richest people [41], the sizes of town and cities [42], the distribution of travelled distances [43, 44] and now, we can also include to this list, the probability of migrating from a city, the probability of moving to the countryside, the probability that an individual from a small city moves to a small city (and the other relationships indicated in Fig 2) and also the size of the city picked as the destination for international migrants. Scaling laws play a fundamental role in the dynamics of migration.

### An improved model of human migration

Our initial scaling model examined human migration without considering the physical distance between cities, that is, only considering the city size. This stance is supported by the data which indicates that individuals from large cities are more likely to move to other large cities, despite the fact that large cities can be far away from each other and relatively scarce. Our gravity-scaling model considers also the impact of distance and so it could be considered a modified version of the gravity model. The gravity-scaling model has a better fit to the observed data, is not symmetric, does not have a systematic bias (as can be observed in the gravity model) and takes into account scaling (from the probability of migrating to the preferential destination picked by migrants).

By considering scaling on migration patterns, the commonly used gravity model is considerably improved, highlighting the relevance of city size. A valuable aspect of both the scaling and the gravity-scaling models is that rather than providing a deterministic number for the flux between two cities, they give a procedure to simulate migration providing intervals which could be observed under the same circumstances. Both models begin by taking into account the number of inhabitants of a city and simulate whether individuals move and, if so, where do they move to. Therefore, there is a natural upper limit to the estimated number of individuals who leave that city, as opposed to the gravity model which might, under certain circumstances, estimate more individuals leaving that actually live there.

### A large city versus a small town

Living in a large city may mean an improved access to education, job opportunities and income, among other “benefits”, but on average and it does not mean better education or income to all; however, the costs of living in a large city is experienced by all its inhabitants. The population living in Kibera, for instance (a slum in Nairobi, Kenya, with approx 1.2 million slum dwellers) or Rocinha (the largest favela of Rio de Janeiro) enjoy a limited number of the benefits of living in a large city but they pay the price for longer commuting distances, a higher price for the food and services, pollution, crime rates and more. Thus, although large cities provide certain benefits, more people moving into large cities does not necessarily translate to people enjoying a better standard of living, but might, unfortunately, translate into greater inequality and severe socio-economic problems within the cities.

The same applies to people from smaller cities. Take, for instance, the case of Carson City, one of the smallest cities in the US, where nearly twice the amount of people moved to Redding than to Sacramento (both in California), even though Sacramento is nearly 100 kilometres closer to Carson City than Redding is and Sacramento is 12 times larger in terms of population size. According to the gravity model, we would expect 20 times more people moving to Sacramento than to Redding since it is larger and closer, but we observe twice the amount of people moved to Redding than to Sacramento; scaling affects migration. Perhaps this is because Redding is a rural environment more similar to Carson City than Sacramento is, although this also warrants further explanation.

### Using only population and distance variables

In terms of migration, not everything can be explained by using only population and distance variables, as used in the models presented here. There are other reasons which create relevant push and pull factors for each city, for instance, Rochester, Minnesota might attract more medical doctors, whilst Ithaca and New Haven might attract more students. Migration is a complex social phenomenon, which begins with individuals deciding whether or not to migrate and develops further when the migrant selects their destination. The mathematical models used here focus on the detection of the patterns which emerge when millions of individuals move from one city to the other.

In particular, the scaling model and the gravity-scaling model show a generalised pattern that is observed with noise being a normal and expected part of the model, as they are a simplification of a much more complex reality. Nonetheless, here results show that scaling is a fundamental aspect of the individual decision of moving and the destination picked by those who decide to move.

### Migration patterns might not be stable

The observed patterns might change and migration to small or large cities might be consequently affected. The current main drivers of migration could subsequently be replaced by other drivers, such as technology, an ageing population, jobs being lost due to automation, climate change, conflicts, fear of crime, water scarcity or other disasters, to name but a few. Cities might have experience positive and negative shocks, which affect their push and pull factors which change how they influence both internal and international migration. The methodology presented here allows different scaling patterns to be detected, through different time intervals, to be applied to international migration or migration from and to the countryside and the detection of quantitative and qualitative changes of human migration.

The methodology presented allows different scaling patterns to be detected, through different time intervals, to be applied to international migration or migration from and to the countryside and the detection of quantitative and qualitative changes in human migration.

The scaling and the gravity-scaling models are based on current observations of migration but it does not mean that the same pattern has been observed previously, nor does it mean that the same pattern will be observed in the future. However, the methodology presented here allows scaling to be taken into account from and to the countryside, between cities and from international migrants and it goes beyond a static result observed only for a specific time interval and a particular region of the world. It highlights that in the studies of migration patterns, scaling might occur, it does so without assuming that scaling happens. In the case in which the exponent *β* ∼ 1 the scaling might be negligible.

### The role of distance in human migration

Although distance does play a crucial role in migration, either because of the mental cost of being far from the origin, the lack of information about distant places [37] or the actual monetary cost of moving, our results indicate that distance could also be expressed in terms of the lifestyle of the individual and not only in terms of physical distance. For example, the four most frequent destinations for an individual who used to live in New York City are Philadelphia, Miami, Washington and Los Angeles, which are 1,800 and 3,900 kilometres away from New York City in the second and fourth case, respectively. Modern communications and rapid transportation mean that the impact of physical distance is reduced so that in terms of migration, distance is becoming less relevant, while the differences in lifestyle between large cities and small cities or countryside are gaining prominence. There are several reasons why the scaling laws affect migration patterns. Our findings suggest that a relevant cause is that an individual chooses between the lifestyle of a large city or the lifestyle of a small one.

### The scaling of international migration

There are still open questions regarding the scaling phenomenon in the case of international migration. Is a person from a large city more likely to move to another country, despite the fact that people from large cities are less likely to migrate? Is the relationship found here, where an individual from a small city is more likely to move to equally small cities, also observed for international migration? Unfortunately, in our dataset there is little information about the origin of international migrants who arrive in the US. However, in terms of their destination we find a strong impact of the city size, which is even more prominent for people who previously lived in Africa or America but outside the US. An individual is less likely to move to a city in which they have less information [37], which might be the reason why people from other countries are more likely to move to a large city.

### The scaling of migration in other parts of the world

Although the results obtained here are based on data for migration to and from cities in the US, a similar scaling pattern is expected in other countries, so that we predict that an individual from Paris is less likely to move to the countryside than a person from Tours, a smaller French city; a person from Guangzhou is more likely to move to Beijing or Shanghai since both cities have millions of inhabitants, even though they are at 1,200 and 1,900 kilometres away respectively; and Sidney, Melbourne and Brisbane are increasing their international population at a faster rate than the rest of Australia. We predict scaling to be relevant for other types of migration and in other regions of the world, although there might be other drivers, for instance, language, weather, conflicts or government-controlled migration policies.

## Tables of results

Results of selected estimated parameters according to different thresholds of city size. The estimation of the parameters uses a logarithmic transformation on both sides of Eq 1 and so the results for $\hat{\alpha }$ are expressed in terms of its natural logarithm. Table 2 has the coefficients obtained for the outflow of internal migration and Table 3 has the coefficients obtained for the inflow of migration to the cities and the countryside in the US.

**Table 2:** Coefficients obtained for the outflow of internal migration in the US.

Outflow of migration (move to…)	$\hat{\beta }$	*s*.*e*.	$\hat{\text{log}\alpha }$	*s*.*e*.	Adj. *R*^2^
Migrating (leaving the city)	0.8829	0.0147	-1.7863	0.1867	0.9033
The countryside	0.6846	0.0273	-0.7214	0.3464	0.6199
City with **less** than 200,000 inhabitants	0.8060	0.0263	-2.8555	0.3325	0.7101
City with **less** than 300,000 inhabitants	0.8199	0.0232	-2.6039	0.2949	0.7636
City with **less** than 400,000 inhabitants	0.8171	0.0222	-2.285	0.2811	0.7794
City with **less** than 500,000 inhabitants	0.8224	0.0206	-2.1614	0.2607	0.8061
City with **less** than 1,000,000 inhabitants	0.8363	0.0175	-1.9163	0.2224	0.8554
City with **less** than 3,000,000 inhabitants	0.8609	0.0159	-1.8352	0.2021	0.8863
City with **less** than 5,000,000 inhabitants	0.8689	0.0157	-1.8189	0.1999	0.8877
City with **more** than 1,000,000 inhabitants	0.9570	0.0211	-3.4759	0.2674	0.8426
City with **more** than 3,000,000 inhabitants	1.0073	0.0307	-4.8421	0.3891	0.7369
City with **more** than 5,000,000 inhabitants	1.0499	0.0337	-5.8437	0.4271	0.7163
City with **more** than 8,000,000 inhabitants	1.1688	0.0506	-8.5108	0.6408	0.5814
City with **more** than 10,000,000 inhabitants	1.2984	0.0619	-10.6921	0.7855	0.5327

**Table 3:** Coefficients obtained for the inflow of migration to the cities and the countryside in the US.

Inflow of migration (moving from…)	$\hat{\beta }$	*s*.*e*.	$\hat{\text{log}\alpha }$	*s*.*e*.	Adj. *R*^2^
The countryside	0.5971	0.0342	0.4299	0.4331	0.4421
City with **less** than 200,000 inhabitants	0.7997	0.0299	-2.7992	0.3799	0.6492
City with **less** than 300,000 inhabitants	0.8036	0.0277	-2.4173	0.3507	0.6869
City with **less** than 400,000 inhabitants	0.8110	0.0262	-2.2685	0.3315	0.7144
City with **less** than 500,000 inhabitants	0.8159	0.0245	-2.1231	0.3103	0.7429
City with **less** than 1,000,000 inhabitants	0.8331	0.0211	-1.9238	0.2678	0.8018
City with **less** than 3,000,000 inhabitants	0.8437	0.0203	-1.6504	0.2570	0.8184
City with **less** than 5,000,000 inhabitants	0.8521	0.0202	-1.6248	0.2555	0.8230
City with **more** than 1,000,000 inhabitants	0.9193	0.0251	-2.8957	0.3177	0.7777
City with **more** than 3,000,000 inhabitants	0.9776	0.0346	-4.2905	0.4390	0.6744
City with **more** than 5,000,000 inhabitants	1.0184	0.0382	-5.2401	0.4851	0.648
City with **more** than 8,000,000 inhabitants	1.1180	0.0460	-7.4146	0.5834	0.6053
City with **more** than 10,000,000 inhabitants	1.2539	0.0574	-9.6531	0.7272	0.5538
International	1.1884	0.0339	-7.9153	0.4305	0.761
Africa	1.5794	0.0728	-16.5087	0.9230	0.55
Asia	1.2207	0.0519	-9.3143	0.6588	0.5891
Americas (outside US)	1.2808	0.0424	-10.5175	0.5374	0.7036
Europe	1.2264	0.0515	-10.2535	0.6530	0.5956
Oceania	1.3312	0.0747	-14.5814	0.9465	0.452
